# Preliminary Insights into the Antigenotoxic Potential of Lemon Essential Oil and Olive Oil in Human Peripheral Blood Mononuclear Cells

**DOI:** 10.3390/plants13121623

**Published:** 2024-06-12

**Authors:** Sara Gonçalves, Mafalda Monteiro, Isabel Gaivão, Rita S. Matos

**Affiliations:** 1 Academic Clinical Center of Trás-os-Montes and Alto Douro—CACTMAD, University of Trás-os-Montes and Alto Douro, 5000-801 Vila Real, Portugal; sgoncalves@utad.pt (S.G.); armatos@arsnorte.min-saude.pt (R.S.M.); 2 Associate Laboratory for Animal and Veterinary Sciences (AL4AnimalS), University of Trás-os-Montes and Alto Douro, 5000-801 Vila Real, Portugal; 3 Centre for Animal Sciences and Veterinary Studies—CECAV, University of Trás-os-Montes and Alto Douro, 5000-801 Vila Real, Portugal; 4 Department of Genetics and Biotechnology, School of Life and Environmental Sciences, University of Trás-os-Montes and Alto Douro, 5000-801 Vila Real, Portugal; 5 Local Health Unit of Trás-os-Montes and Alto Douro, 5050-275 Peso da Régua, Portugal

**Keywords:** lemon essential oil, olive oil, antigenotoxicity, comet assay, streptonigrin

## Abstract

Lemon essential oil, derived from *Citrus limon*, possesses diverse health-promoting properties, including antioxidant, antimicrobial, and mood-enhancing effects. Despite its traditional use in aromatherapy and complementary medicine, there is a need for comprehensive investigations into its therapeutic potential, particularly in mitigating DNA damage and supporting health in palliative care settings. This study aimed to evaluate the antigenotoxic effects of lemon essential oil in human peripheral blood mononuclear cells and to explore its potential applications in palliative care. Treatment with lemon essential oil significantly reduced DNA damage, with 1% w/v with 3.13% DNA in tail demonstrating greater efficacy. Furthermore, lemon essential oil attenuated streptonigrin-induced DNA damage, suggesting a potential protective effect against oxidative stress, especially at 3% w/v, with 11.81% DNA in tail. Compared to olive oil treatment, the DNA damage was significantly lower with streptonigrin treatment alone, which had 47.06% DNA in tail, while the olive oil treatment resulted in 36.88% DNA in tail. These results can be attributed to the main constituents: limonene in lemon essential oil and oleic acid in olive oil. These results suggest a potential role in mitigating oxidative stress and supporting genomic stability. Further research is warranted to elucidate the mechanisms of action and clinical applications in palliative care.

## 1. Introduction

In recent years, there has been growing interest in exploring natural compounds for their potential therapeutic benefits in addressing various health conditions [1]. Plants have always been crucial sources of medicinal compounds, and their traditional use in diverse cultures has been valued for centuries. Modern research increasingly recognises the effectiveness of plant extracts in preventing and treating various diseases, including antioxidant, anti-inflammatory, antimicrobial, and anticancer properties [2,3]. Harnessing the therapeutic potential of plants and their extracts can pave the way for developing novel drugs, functional foods, and nutraceuticals [4,5]. Among these, essential oils have garnered attention for their diverse pharmacological properties [6].

Lemon essential oil, derived from the peel of *Citrus limon*, is a natural product traditionally used for its medicinal properties and aromatic qualities [7]. Rich in bioactive constituents, such as limonene, citral, and linalool, lemon essential oil has been studied for its potential health-promoting effects in areas ranging from skincare to mental health [8]. In addition to its antioxidant effects, lemon essential oil exhibits antimicrobial properties, making it a valuable agent for combating bacterial, fungal, and viral infections. Studies have shown that lemon essential oil may inhibit the growth of bacteria, such as *Staphylococcus aureus* and *Escherichia coli*, as well as fungi like *Candida albicans* [9,10]. These antimicrobial properties lend themselves to various applications, including topical treatments for skin infections and oral care products for maintaining oral hygiene.

Olive oil (*Olea europaea*), another plant-derived compound, has also been extensively studied for its health benefits [11]. Rich in monounsaturated fatty acids, mainly oleic acid, and various antioxidants, olive oil is well known for its anti-inflammatory and cardioprotective properties [12]. Its consumption is a vital component of the Mediterranean diet, and is associated with reduced risks of cardiovascular diseases, certain cancers, and overall mortality [13]. Beyond its dietary benefits, olive oil’s bioactive compounds have been shown to promote genomic stability and protect against oxidative stress [14]. This makes olive oil a valuable substance not only for nutritional purposes but also for therapeutic applications, including its potential role in supporting immune function and reducing inflammation [15].

However, despite the growing body of evidence supporting the biological activities of lemon essential oil, there remains a need for comprehensive investigations into its mechanisms of action, therapeutic potential, and clinical applications. Recent research has highlighted the role of oxidative stress in DNA damage, which is implicated in ageing and various diseases, including cancer [16]. Essential oils, with their antioxidant properties, could significantly mitigate oxidative stress and protect genomic stability [17]. Therefore, this study aims to evaluate the antigenotoxic effects of lemon essential oil in human peripheral blood mononuclear cells and explore its implications for palliative care, particularly in alleviating symptoms such as nausea and vomiting. By elucidating the protective effects of lemon essential oil against DNA damage and oxidative stress, this research seeks to contribute to the understanding of natural compounds as adjunctive therapies in promoting health and well-being.

## 2. Results

This study assessed DNA damage in human peripheral blood mononuclear cells (PBMCs) using a Comet assay to investigate the impact of lemon essential oil concentrations and streptonigrin (SN) challenges.

**Figure 1:**
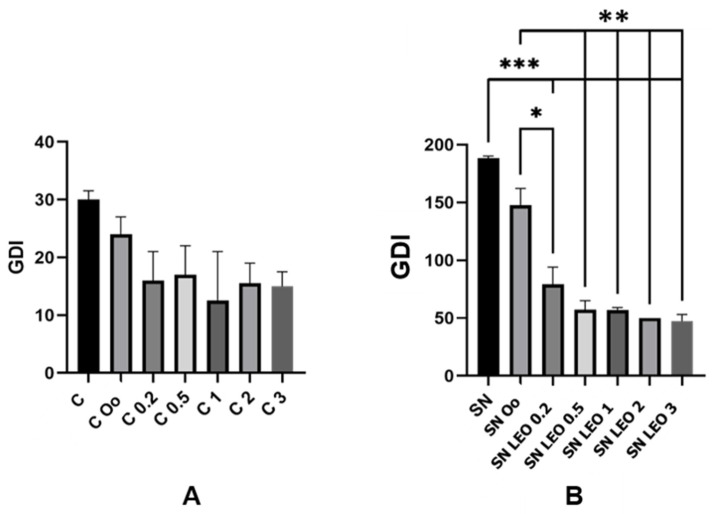
Assessment of the genetic damage indicator (GDI) in human PBMCs. The mean values of DNA damage, quantified as arbitrary units using the in vivo Comet assay, were determined in both the unchallenged and SN-challenged groups. (**A**) the unchallenged group and (**B**) the SN-challenged group. The ‘C’ designation corresponds to the control group treated exclusively with PBS. The designation ‘C Oo’ corresponds to the control solely treated with olive oil. The designations ‘C 0.2’, ‘C 0.5’, ‘C 1’, ‘C 2’, and ‘C 3’ correspond to olive oil treatment and the respective lemon essential oil concentrations (0.2: 0.2%, 0.5: 0.5%, 1: 1%, 2: 2% and 3: 3%). ‘SN’ signifies the group subjected solely to SN treatment. The designation ‘SN Oo’ corresponds to the SN treated exclusively with olive oil. The tested groups are distinguished by abbreviations denoting the constituent ingredient (LEO: lemon essential oil) and the respective lemon essential oil concentrations (0.2: 0.2%, 0.5: 0.5%, 1: 1%, 2: 2%, and 15: 15%). The single asterisk stands for significant differences between the olive oil treatment and LEO treatment of 0.2%. The double asterisk stands for significant differences between the olive oil and LEO treatments of 0.5%, 1%, 2% and 3%. The triple asterisk stands for significant differences between the control treatment and all LEO treatments. The error bars represent standard errors.

In the unchallenged groups, PBMCs treated solely with lemon essential oil (LEO) exhibited a concentration-dependent decrease in DNA damage compared to the untreated controls. Specifically, cells treated with higher concentrations of LEO demonstrated significantly reduced DNA damage, as indicated by the lower mean values of arbitrary units measured by the Comet assay. Among the unchallenged groups, LEO at a concentration of 1% exhibited the most pronounced reduction in DNA damage (Figure 1).

Furthermore, co-treatment with LEO and SN showed a mitigating effect on SN-induced DNA damage in PBMCs. The combination of LEO with SN resulted in lower mean values of DNA damage compared to cells treated solely with SN. Notably, the extent of DNA damage reduction appeared to correlate with the concentration of LEO. Among the SN-challenged groups, LEO at a concentration of 3% exhibited the most significant decrease in DNA damage.

Table 1 illustrates the percentage of DNA in the tail (% DNA in tail) for various treatments, indicating the level of DNA damage. Lower values represent reduced DNA damage, demonstrating the protective effects of lemon essential oil and olive oil against oxidative stress, as measured by the percentage of DNA in the tail. The control group (C) exhibited a % DNA in tail of 7.50%, while the control group treated with olive oil (C Oo) showed a reduction to 6.00%. Different concentrations of lemon essential oil demonstrated a further reduction in DNA damage, with the lowest % DNA in tail observed at 1% w/v (C 1) with 3.13%. Higher concentrations of LEO (2% and 3% w/v) also showed significant reductions to 3.88% and 3.75%, respectively.

A significant increase in DNA damage was observed for cells exposed to SN, with a % DNA in tail of 47.06%. However, treatment with olive oil (SN Oo) reduced this damage to 36.88%. Lemon essential oil treatments at various concentrations (SN LEO 0.2 to SN LEO 3) resulted in a substantial decrease in DNA damage. The lowest DNA damage in the streptonigrin-treated groups was observed with 3% w/v LEO (SN LEO 3), which had a % DNA in tail of 11.81%, followed closely by 2% w/v LEO (SN LEO 2) at 12.50%. These findings indicate that both olive oil and lemon essential oil possess antigenotoxic properties, with lemon essential oil demonstrating greater efficacy in reducing DNA damage, particularly at higher concentrations.

## 3. Discussion

The observed significant reduction in DNA damage following treatment with lemon essential oil can be attributed to its chemical composition, particularly limonene. Limonene, a monocyclic monoterpene, constitutes a substantial portion of lemon essential oil, as evidenced by the chemical characterisation of our lemon essential oil, revealing its presence at 68.13% (Supplementary Material–Figure S1). Limonene is well documented for its potent antioxidant properties, which can scavenge reactive oxygen species and inhibit DNA damage induced by oxidative stress [18,19]. As a natural antioxidant, limonene may mitigate DNA damage by neutralising free radicals and preventing oxidative modifications to DNA bases. Additionally, limonene possesses anti-inflammatory properties, which may further contribute to its protective effects against DNA damage by attenuating inflammatory responses and oxidative stress pathways [20]. Therefore, the abundance of limonene in lemon essential oil likely underlies its ability to reduce DNA damage in human PBMCs and highlights its potential as a therapeutic agent for combating disorders induced by oxidative stress. Further research elucidating the mechanistic pathways involved in the protective effects of limonene on DNA integrity is warranted to fully understand its therapeutic potential.

The observed effects of olive oil on DNA damage may be attributed to its chemical composition, particularly its high oleic acid content. The chemical characterisation of our olive oil revealed that olive oil contains approximately 70.77% oleic acid (Supplementary Material–Figure S2). Oleic acid, a monounsaturated omega-9 fatty acid, is renowned for its beneficial health effects and its anti-inflammatory and antioxidant properties [21,22]. While the specific mechanisms underlying its effects on DNA damage are not yet fully elucidated, oleic acid exerts protective effects against damage induced by oxidative stress by scavenging free radicals and modulating inflammatory pathways [23]. Furthermore, oleic acid has been implicated in regulating the cellular signalling pathways involved in DNA repair and the maintenance of genomic stability [24,25]. Therefore, a high proportion of oleic acid in olive oil likely contributes to its ability to attenuate DNA damage independently and in combination with other agents. Further research is warranted to unravel the precise molecular mechanisms through which oleic acid confers its protective effects on DNA integrity and to explore its potential therapeutic applications in preventing diseases related to DNA damage. The results obtained with olive oil treatment closely align with findings from a previous study, indicating consistent outcomes across independent investigations [26].

The five concentrations of lemon essential oil were selected based on their relevance in palliative care [27]. Palliative care focuses on improving the quality of life for individuals with severe illnesses by addressing their physical, emotional, and spiritual needs. Essential oils, including lemon essential oil, are increasingly recognised for their potential therapeutic benefits in palliative care, particularly in managing symptoms such as nausea and vomiting in various populations, including cancer patients undergoing chemotherapy and individuals with terminal illnesses [28]. The chosen concentrations of lemon essential oil (0.2%, 0.5%, 1%, 2%, and 3%) represent a range commonly used in aromatherapy and complementary medicine practices within palliative care settings. These concentrations have been reported to be well tolerated by patients and are considered safe for topical and inhalation use [29]. 

By including a range of concentrations, from lower to higher doses, our study aimed to explore the dose–response relationship of lemon essential oil in mitigating DNA damage. This approach allows the assessment of the safety and efficacy profiles of lemon essential oil across different concentrations, aiming to optimise its therapeutic potential in palliative care. Furthermore, using concentrations commonly employed in clinical practice enhances the translational relevance of our findings and facilitates the integration of interventions based on lemon essential oil into palliative care protocols.

In palliative care, patients often face heightened oxidative stress due to factors like disease progression, chemotherapy, or radiation therapy. By counteracting DNA damage induced by oxidative stress, lemon essential oil could indirectly alleviate symptoms such as nausea, which can worsen with systemic inflammation and oxidative stress. Beyond its antioxidant properties, limonene, a key component of lemon essential oil, exhibits anti-inflammatory effects. Inflammation commonly underlies various symptoms in palliative care, including nausea. Lemon essential oil may relieve nausea linked to inflammatory conditions or treatments by dampening inflammatory responses and oxidative stress pathways. Palliative care emphasises holistic symptom management to enhance patients’ quality of life amidst life-limiting illnesses. While our study primarily investigated lemon essential oil’s antigenotoxic effects, its broader antioxidant and anti-inflammatory properties resonate with the holistic palliative care approach. By targeting underlying factors like oxidative stress and inflammation, lemon essential oil can potentially alleviate symptoms, including nausea, in palliative patients.

The lemon essential oil used in this study was intentionally selected for its absence of furanocoumarins, compounds known for their potential phototoxic effects [30]. This decision prioritised participant safety, especially considering potential sunlight or UV exposure. Looking ahead, future research aims to extend this investigation into palliative care settings, where lemon essential oil could be applied to alleviate symptoms such as nausea and vomiting. This expansion promises to shed light on the antigenotoxic effects of lemon essential oil in palliative care patients, offering insights into its therapeutic potential in addressing common symptoms associated with severe illnesses.

Given its accessibility, affordability, and favourable safety profile, lemon essential oil holds promise as a complementary therapy for controlling nausea and vomiting in palliative care settings. Integrating lemon essential oil into holistic care plans addresses physical symptoms and contributes to the overall well-being and comfort of patients facing life-limiting illnesses.

In future investigations, it would be valuable to explore the antigenotoxicity of lemon essential oil, specifically within the context of palliative care patients. Given the unique physiological and psychological challenges faced by individuals receiving palliative care, understanding the potential protective effects of lemon essential oil against DNA damage in this population is crucial. By conducting comparative studies between palliative care patients and healthy individuals, researchers can elucidate any differential responses to lemon essential oil treatment, considering factors such as disease status, medication use, and overall health status. This research endeavour holds promise for uncovering novel therapeutic strategies to mitigate DNA damage and improve the quality of life for individuals navigating end-of-life care.

### Limitations

This study is not without limitations, which should be acknowledged to provide a comprehensive understanding of the research findings and guide future investigations.

One significant limitation is the reliance on peripheral blood samples from a single healthy volunteer. While this approach allowed for controlled experimental conditions, it restricted the generalisability of the findings to a broader population. Moreover, using blood from a single volunteer fails to account for potential variations in DNA damage responses related to sex, age, genetic background, or health status.

The in vitro experimental design using human PBMCs provides valuable insights into cellular responses, but may not fully replicate the complex interactions occurring in a living organism. In vivo studies must validate these findings in a more physiologically relevant context.

Furthermore, the study focused on specific concentrations of lemon essential oil, chosen based on their relevance in palliative care. Exploring a wider range of concentrations, including those outside typical therapeutic ranges, might offer a better understanding of the dose–response relationship and potential adverse effects.

While limonene and oleic acid were identified as the major constituents of lemon essential oil and olive oil, respectively, this study did not isolate their individual effects. Future research should include comparative experiments with pure limonene and oleic acid to determine whether the observed antigenotoxic effects are attributable to these main components or to the mixture of components present in the oils.

Additionally, the study attributes the protective effects of lemon essential oil to its antioxidant properties, particularly limonene, without directly measuring the antioxidant capacity of the essential oil in the experimental setup. Including assays for antioxidant activity (e.g., DPPH, ABTS) could strengthen this claim.

The study does not provide direct evidence of the underlying mechanisms responsible for the observed effects. Future research should include mechanistic studies to elucidate the specific pathways involved in DNA repair and oxidative stress response.

Lastly, while the study discusses the potential application of lemon essential oil in palliative care, it does not provide empirical data on its efficacy in clinical settings. Clinical trials are needed to evaluate the safety and effectiveness of lemon essential oil in palliative care patients, considering its potential long-term effects and practical implications.

## 4. Materials and Methods

### 4.1. Cells

Peripheral blood samples were obtained from one healthy volunteer, a 36-year-old female. The volunteer was non-smoking, non-alcoholic, had no health problems, and had not taken any medication for six months before this study. This research was performed with the permission of the Ethical Committee for Health of the Local Health Unit of Trás-os-Montes and Alto Douro (30/2024-CES) and under the Declaration of Helsinki.

### 4.2. Chemicals

Streptonigrin (CAS 3930–19-6) was purchased from Santa Cruz Biotechnology Inc. (Dallas, TX, USA). Lemon essential oil (INCI: *Citrus limon Peel Oil*; lot 23HE0041/2) was purchased from AromaZone (Paris, France). The essential oil was extracted from the zest (pericarp) of *Citrus limon* using a combination of cold pressing and steam distillation to remove furocoumarins. This oil is 100% pure, natural, and food-grade, certified organic by Ecocert FR-BIO-01, and botanically and biochemically defined (HEBBD). The lemons used were grown in Sicily, Italy. Olive oil (INCI: *Olea europaea*; lot 24-2307-63) was obtained from Cooperativa Agrícola dos Olivicultores de Murça, CRL (Murça, Portugal). All other chemicals were purchased from Sigma-Aldrich Chemical Company (Madrid, Spain).

### 4.3. Lysis Solution

The lysis solution underwent meticulous formulation, combining 2.5 M NaCl, 0.1 M EDTA disodium salt, and 0.01 M Tris base, with the pH adjusted to 10. Initially, a solution devoid of Triton X-100 was prepared in distilled water, slightly below the final volume, with precise amounts of each component. Subsequently, pH adjustment to 10 occurred gradually over an hour at 4 °C using a 10 M NaOH solution. Lastly, 1% Triton X-100 was added to the lysis solution just before its application.

### 4.4. Phosphate-Buffered Saline (PBS) Solution

A PBS solution was prepared by dissolving exact amounts of 2 mM KH_2_PO_4_, 10 mM Na_2_HPO_4_, 2.7 mM KCl, and 137 mM NaCl in distilled water, slightly below the final required volume. The pH was carefully adjusted to 7.4 using a 1 M HCl solution. Then, the remaining water necessary to reach the final volume was added to complete the PBS solution.

### 4.5. Electrophoresis Solution

The electrophoresis solution was prepared by mixing 0.3 M NaOH and 1 mM EDTA in a flask. Distilled water was then gradually added until the pH reached approximately 12.6. The pH was monitored using a calibrated pH meter to ensure consistency. Adjustments were made as necessary by adding small amounts of either NaOH or EDTA solution. This process was repeated until the desired pH was achieved. The final solution was then filtered to remove any particulate matter and stored in a clean, labelled container until further use.

### 4.6. Lemon Essential Oil and Olive Oil Treatments

For the Comet assay, five concentrations of lemon essential oil were chosen based on the previous literature review: 0.2%, 0.5%, 1%, 2%, and 3% (w/v) [27]. These concentrations were utilised in two distinct treatments: one in olive oil alone and the other in a mixture of olive oil and 20 μM of SN. Two independent experiments, 10 days apart, were performed for each condition.

SN treatment cultivation involved dissolving SN in PBS to attain a final concentration of 20 μM within a 5 mL volume, following established methodologies [31]. The experiment involved setting up 14 slides: the first held only PBS, the second held olive oil, and the third to seventh each contained different lemon essential oil concentrations (0.2%, 0.5%, 1%, 2%, or 3% (w/v) combined with olive oil. The eighth held only the SN-treated solution, the ninth held only olive oil, and the tenth to fourteenth contained various lemon essential oil concentrations combined with the SN treatment.

**Figure 2:**
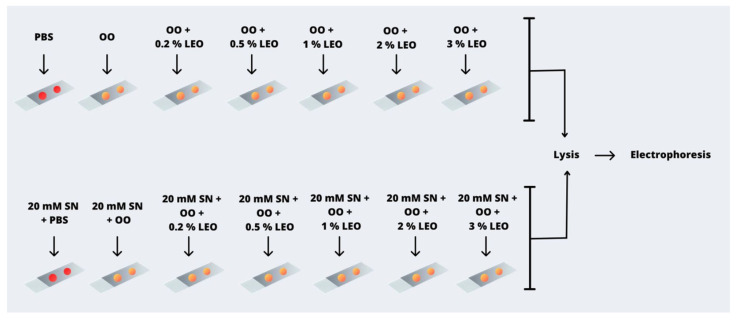
Illustration of the SN treatment process: 14 slides were used in this setup. After collecting a blood sample via finger prick, the gel matrix was treated with various treatments. The first contained only PBS, the second olive oil (OO), and the third to the seventh contained different concentrations of lemon essential oil (LEO) combined with OO. The eighth slide held a combination of PBS and SN, the ninth a combination of OO and SN, and the remaining slides accommodated a blend of OO, SN, and distinct lemon essential oil concentrations (0.2%, 0.5%, 1%, 2%, and 3%). All slides followed conventional procedures involving lysis and electrophoresis.

The detailed workflow is visually represented in Figure 2, which provides an illustrative overview.

### 4.7. Genotoxic Evaluation

The genotoxic/antigenotoxic effects of lemon essential oil and olive oil were evaluated through a Comet assay in vivo in human PBMCs.

#### Comet Assay in Human PBMCs Using SN

The experimentation followed the outlined methodology [31]. All solutions and precoated slides containing 1% normal melting point agarose were prepared before the experimental session. Blood samples were collected through finger pricks, with 25 µL of each sample applied onto 0.8% low melting point agarose in PBS. Subsequently, two 70 µL drops of this mixture were deposited onto precoated slides, each covered with a coverslip to ensure uniform dispersion. This process was replicated for each concentration under investigation. Slides were refrigerated at 4 °C for 5 min to solidify the agarose before removing the coverslips. Following this, the slides underwent various treatment concentrations with lemon essential oil dissolved in a 50 µL droplet of a mixture of SN and olive oil placed on the agarose gel and the blood sample that was then covered with a coverslip. Then, they were incubated at 37 °C for 1 h and immersed in a cold, fresh lysis solution.

Arranged without gaps in the electrophoresis chamber, the slides were submerged in a cold denaturing and electrophoresis buffer for 20 min. Electrophoresis was carried out in darkness at 4 °C with a current of 300 mA and a voltage of 25 V (equivalent to 0.8 V/cm) for 20 min. Post-electrophoresis, slides were washed sequentially in PBS (10 min at 4 °C) and distilled water (10 min at 4 °C) before being air-dried.

Each gel was then stained with 40 μL of DAPI (4′,6-diamidino-2-phenylindole) (1 μg/mL in dH_2_O) and covered with a coverslip for examination under a fluorescence microscope (Leica DMLS fluorescence microscope) at 400x magnification. Fifty cells per gel were observed, and the tail intensity of each cell was graded from 0 (no tail) to 4 (almost all DNA in the tail) [32]. Finally, the final score, expressed in arbitrary units ranging from 0 to 400, was computed using the genetic damage indicator (GDI) formula.

$$\begin{array}{c} Genetic\;Damage\;Indicator\;\left(GDI\right)\\ \;\;=[(\%\;nucleoid\;class\;0)\times 0)]+[(\%\;nucleoid\;class\;1)\times 1)]+[(\%\;nucleoid\;class\;2)\times 2)]\\ \;\;+[(\%\;nucleoid\;class\;3)\times 3)]+[(\%\;nucleoid\;class\;4)\times 4)] \end{array}$$

Embedding cells in agarose gel for the Comet assay is essential to immobilise the cells and preserve their structural integrity throughout the procedure. This technique is beneficial for analysing cells that grow in adherent monolayers, as it simplifies their handling and manipulation [33]. The agarose gel creates a supportive matrix that encapsulates the cells, preventing them from becoming dislodged or damaged during the various stages of the assay, such as cell lysis and electrophoresis. This encapsulation ensures an even distribution of cells on microscope slides, allowing for consistent analysis [34].

The porous nature of agarose also facilitates the diffusion of lysis solutions and other reagents, which helps maintain cell integrity while efficiently removing proteins and contaminants during the lysis process. This enhancement improves the visualisation and analysis of DNA damage [33,35]. For cells in suspension, embedding in agarose is crucial for accurate Comet assay analysis. Without embedding, suspended cells would disperse during electrophoresis, making it challenging to assess DNA damage levels accurately. By embedding these cells in agarose, they are immobilised, ensuring reliable and consistent DNA damage analysis throughout the assay steps [36,37].

### 4.8. Statistical Analysis

Data were analysed using IBM SPSS statistics software (Statistical Package for the Social Sciences, Chicago, IL, USA), version 20. An analysis of variance (ANOVA) was performed, followed by a Tukey test. Differences were considered statistically significant if *p* < 0.05.

## 5. Conclusions

Our study has provided valuable insights into the potential therapeutic effects of lemon essential oil and olive oil in mitigating DNA damage. By assessing DNA damage in human PBMCs using the Comet assay, we have demonstrated that lemon essential oil and olive oil exhibit concentration-dependent protective effects against DNA damage, independently and combined with the DNA-damaging agent SN. Specifically, lemon essential oil showed significant attenuation of SN-induced DNA damage, highlighting its potential as a protective agent against oxidative stress. Similarly, olive oil also displayed protective properties, suggesting its beneficial role in reducing DNA damage.

These results underscore the importance of further exploring the mechanisms underlying lemon essential oil’s antioxidant and antigenotoxic properties, particularly in palliative care, where its application may offer therapeutic benefits in alleviating symptoms such as nausea and vomiting.

Beyond palliative care, the findings have broader implications for using natural products in preventive health strategies and chronic disease management. The potential of lemon essential oil and olive oil to protect against oxidative DNA damage may reduce the risk of diseases linked to oxidative stress, such as cancer, cardiovascular diseases, and neurodegenerative disorders. Furthermore, incorporating these oils into daily dietary practices could provide a complementary approach to enhancing overall health and well-being.

Our study contributes to the growing body of evidence supporting the potential utility of lemon essential oil and olive oil as natural therapeutic agents for mitigating DNA damage. This warrants further investigation into their clinical applications in palliative care, preventive health, and beyond. Understanding the broader impact of these findings could lead to the development of new dietary recommendations and therapeutic strategies to leverage these natural products’ health benefits.

## Figures and Tables

**Table 1:** Antigenotoxic effects of lemon essential oil and olive oil on DNA damage in human peripheral blood mononuclear cells.

Treatment	GDI	% DNA in Tail
C	30.00	7.50
C Oo	24.00	6.00
C 0.2	16.00	4.00
C 0.5	17.00	4.25
C 1	12.50	3.13
C 2	15.50	3.88
C3	15.00	3.75
SN	188.25	47.06
SN Oo	147.50	36.88
SN LEO 0.2	73.25	18.31
SN LEO 0.5	57.25	14.31
SN LEO 1	57.00	14.25
SN LEO 2	50.00	12.50
SN LEO 3	47.25	11.81

## Data Availability

Data supporting the findings and conclusions are available upon request from the corresponding author.

## Supplementary Materials

The following supporting information can be downloaded at: https://www.mdpi.com/article/10.3390/plants13121623/s1, Figure S1: Chemical characterisation of lemon essential oil provided by Aroma-Zone, Paris, France; Figure S2: Chemical characterisation provided by Cooperativa Agrícola dos Olivicultores de Murça, CRL, Murça, Portugal.
