# Supplementary material for: Preliminary Insights into the Antigenotoxic Potential of Lemon Essential Oil and Olive Oil in Human Peripheral Blood Mononuclear Cells
Source: Plants (Basel). 2024 Jun 12;13(12):1623. doi: 10.3390/plants13121623 (PMC11207684; doi:10.3390/plants13121623)
Supplement: Supplementary file 1 [file plants-13-01623-s001.zip › plants-3048807-supplementary.pdf]

Supplementary Material

AROMA-ZONE
EXPERT NATUREL EN SOINS & BEAUTÉ

Bulletin d'Analyse

**Article contrôlé**	HE0208	HE Citron sans furocoumarines BIO			
**Lot**	23HE0041	**DLU**	31/10/2024	**Date obtention**	01/10/2022
**Origine**	ITALIE	**Mode de culture**	Biologique		

Contrôle effectué	Methode	Unité	Résultat de la mesure
Aspect	QUA_ITMC_015		CONFORME
Couleur	QUA_ITMC_015		CONFORME
Odeur	QUA_ITMC_015		CONFORME
Densite	QUA_ITMC_001		0.85
Indice de peroxyde -mmol/l	QUA_ITMC_044		11.7
Limonene	QUA_ITMC_031	%	68.13
Beta-pinene	QUA_ITMC_031	%	11.15
Gamma-terpinene	QUA_ITMC_031	%	9.42
Alpha-pinene	QUA_ITMC_031	%	1.51
Sabinene	QUA_ITMC_031	%	1.6
Geranial	QUA_ITMC_031	%	1.57
Myrcene	QUA_ITMC_031	%	1.49
Oxypeucedanine	QUA_ITMC_031	%	0
Citroptene	QUA_ITMC_031	%	0
Bergaptene	QUA_ITMC_031	%	0
Bergamottine	QUA_ITMC_031	%	0
Paracymene	QUA_ITMC_031	%	0.52
Safrol	QUA_ITMC_031	%	0
Methyleugenol	QUA_ITMC_031	%	0

ACCEPTE

Le : **15/05/2023**

Validé par : Laboratoire Controle Qualite AROMA-ZONE

Ce certificat est généré automatiquement - Une signature n'est pas requise. L'utilisateur est seul responsable d'être en conformité avec la réglementation applicable relative à la fabrication, transformation, vente, et usages du produit. Les analyses mentionnées dans le présent certificat sont données à titre indicatif comme description du produit. Ces informations n'engagent en aucun cas notre responsabilité juridique concernant les caractéristiques et applications du produit.

AROMA-ZONE - La Cigalette - 1366 Route de Gordes - Le Coustellet - 84220 CABRIERES D'AVIGNON

1

Figure S1. Chemical charcterisation of lemon essential oil provided by Aroma-Zone, Paris, France

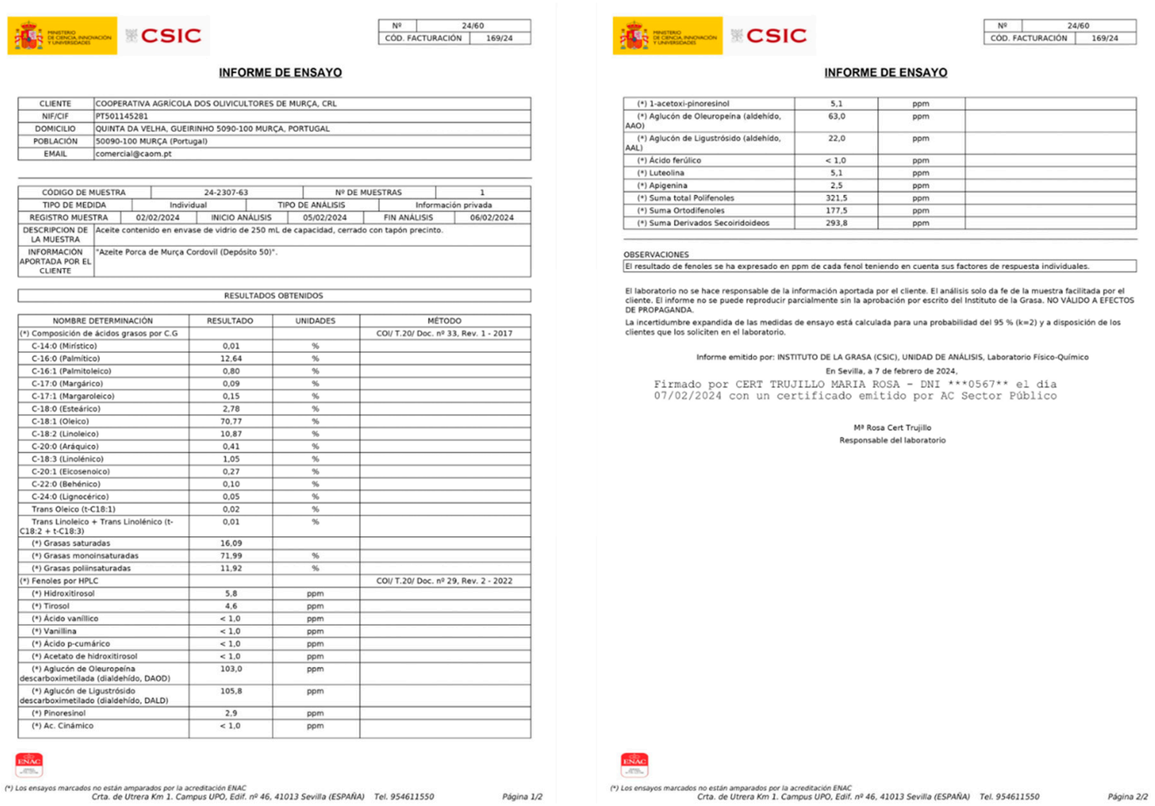

Nº	24/60
CÓD. FACTURACIÓN	169/24

INFORME DE ENSAYO

CLIENTE	COOPERATIVA AGRÍCOLA DOS OLIVICULTORES DE MURÇA, CRL
NIF/CIF	PT501145281
DOMICILIO	QUINTA DA VELHA, GUEIRINHO 5090-100 MURÇA, PORTUGAL
POBLACIÓN	50090-100 MURÇA (Portugal)
EMAIL	comercial@caom.pt

CÓDIGO DE MUESTRA	24-2307-63		Nº DE MUESTRAS	1	
TIPO DE MEDIDA	Individual		TIPO DE ANÁLISIS	Información privada	
REGISTRO MUESTRA	02/02/2024	INICIO ANÁLISIS	05/02/2024	FIN ANÁLISIS	06/02/2024
DESCRIPCION DE LA MUESTRA	Aceite contenido en envase de vidrio de 250 mL de capacidad, cerrado con tapón precinto.				
INFORMACIÓN APORTADA POR EL CLIENTE	"Azeite Porca de Murça Cordovil (Depósito 50)".				

RESULTADOS OBTENIDOS

NOMBRE DETERMINACIÓN	RESULTADO	UNIDADES	MÉTODO
(*) Composición de ácidos grasos por C.G			COI/ T.20/ Doc. nº 33, Rev. 1 - 2017
C-14:0 (Mirístico)	0,01	%	
C-16:0 (Palmítico)	12,64	%	
C-16:1 (Palmitoleico)	0,80	%	
C-17:0 (Margárico)	0,09	%	
C-17:1 (Margaroleico)	0,15	%	
C-18:0 (Esteárico)	2,78	%	
C-18:1 (Oleico)	70,77	%	
C-18:2 (Linoleico)	10,87	%	
C-20:0 (Aráquico)	0,41	%	
C-18:3 (Linolénico)	1,05	%	
C-20:1 (Eicosenoico)	0,27	%	
C-22:0 (Behénico)	0,10	%	
C-24:0 (Lignocérico)	0,05	%	
Trans Oleico (t-C18:1)	0,02	%	
Trans Linoleico + Trans Linolénico (t-C18:2 + t-C18:3)	0,01	%	
(*) Grasas saturadas	16,09		
(*) Grasas monoinsaturadas	71,99	%	
(*) Grasas poliinsaturadas	11,92	%	
(*) Fenoles por HPLC			COI/ T.20/ Doc. nº 29, Rev. 2 - 2022
(*) Hidroxitirosol	5,8	ppm	
(*) Tirosol	4,6	ppm	
(*) Ácido vanillico	< 1,0	ppm	
(*) Vanillina	< 1,0	ppm	
(*) Ácido p-cumárico	< 1,0	ppm	
(*) Acetato de hidroxitirosol	< 1,0	ppm	
(*) Aglucón de Oleuropeína descarboximetilada (dialdehído, DAOD)	103,0	ppm	
(*) Aglucón de Ligustrósido descarboximetilado (dialdehído, DALD)	105,8	ppm	
(*) Pinoresinol	2,9	ppm	
(*) Ac. Cinámico	< 1,0	ppm	

(*) Los ensayos marcados no están amparados por la acreditación ENAC

Crta. de Utrera Km 1. Campus UPO, Edif. nº 46, 41013 Sevilla (ESPAÑA) Tel. 954611550 Página 1/2

Nº	24/60
CÓD. FACTURACIÓN	169/24

INFORME DE ENSAYO

(*) 1-acetoxi-pinoresinol	5,1	ppm	
(*) Aglucón de Oleuropeína (aldehído, AAO)	63,0	ppm	
(*) Aglucón de Ligustrósido (aldehído, AAL)	22,0	ppm	
(*) Ácido ferúlico	< 1,0	ppm	
(*) Luteolina	5,1	ppm	
(*) Apigenina	2,5	ppm	
(*) Suma total Polifenoles	321,5	ppm	
(*) Suma Ortodifenoles	177,5	ppm	
(*) Suma Derivados Secoiridoideos	293,8	ppm	

OBSERVACIONES

El resultado de fenoles se ha expresado en ppm de cada fenol teniendo en cuenta sus factores de respuesta individuales.

El laboratorio no se hace responsable de la información aportada por el cliente. El análisis solo da fe de la muestra facilitada por el cliente. El informe no se puede reproducir parcialmente sin la aprobación por escrito del Instituto de la Grasa. NO VÁLIDO A EFECTOS DE PROPAGANDA.

La incertidumbre expandida de las medidas de ensayo está calculada para una probabilidad del 95 % (k=2) y a disposición de los clientes que los soliciten en el laboratorio.

Informe emitido por: INSTITUTO DE LA GRASA (CSIC), UNIDAD DE ANÁLISIS, Laboratorio Físico-Químico

En Sevilla, a 7 de febrero de 2024.

Firmado por CERT TRUJILLO MARIA ROSA - DNI ***0567** el día 07/02/2024 con un certificado emitido por AC Sector Público

Mª Rosa Cert Trujillo

Responsable del laboratorio

(*) Los ensayos marcados no están amparados por la acreditación ENAC

Crta. de Utrera Km 1. Campus UPO, Edif. nº 46, 41013 Sevilla (ESPAÑA) Tel. 954611550 Página 2/2

Figure S2. Chemical characterisation provided by Cooperativa Agrícola dos Olivicultores de Murça, CRL, Murça, Portugal
